# Active-Q: Validation of the Web-Based Physical Activity Questionnaire Using Doubly Labeled Water

**DOI:** 10.2196/jmir.1974

**Published:** 2012-02-15

**Authors:** Stephanie Erika Bonn, Ylva Trolle Lagerros, Sara Elisabeth Christensen, Elisabeth Möller, Antony Wright, Arvid Sjölander, Katarina Bälter

**Affiliations:** ^1^Department of Medical Epidemiology and BiostatisticsKarolinska InstitutetStockholmSweden; ^2^Clinical Epidemiology UnitDepartment of MedicineKarolinska InstitutetStockholmSweden; ^3^MRCHuman Nutrition ResearchCambridgeUnited Kingdom

**Keywords:** activity assessment, epidemiology, Internet, total energy expenditure

## Abstract

**Background:**

Increased use of the Internet provides new opportunities for collecting data in large studies. The aim of our new Web-based questionnaire, Active-Q, is to assess total physical activity and inactivity in adults. Active-Q assesses habitual activity during the past year via questions in four different domains: (1) daily occupation, (2) transportation to and from daily occupation, (3) leisure time activities, and (4) sporting activities.

**Objective:**

The objective of our study is to validate Active-Q’s energy expenditure estimates using the doubly labeled water (DLW) method, and to assess the reproducibility of Active-Q by comparing the results of the questionnaire completed by the same group on two occasions.

**Methods:**

The validity and reproducibility of Active-Q were assessed in a group of 37 individuals, aged 20 to 65 years. Active-Q was distributed via email to the participants. The total energy expenditure of the participants was assessed using DLW for 11 consecutive days.

**Results:**

The median time to complete Active-Q was 6.1 minutes. The majority of participants (27/37, 73%) reported that the questionnaire was “easy” or “very easy” to answer. On average, Active-Q overestimated the total daily energy expenditure by 440 kJ compared with the DLW. The Spearman correlation between the two methods was r = 0.52 (P < .001). The intraclass correlation coefficient for total energy expenditure between the results of Active-Q completed on two occasions was 0.83 (95% CI 0.73-0.93).

**Conclusions:**

Active-Q is a valid and reproducible method of assessing total energy expenditure. It is also a user-friendly method and suitable for Web-based data collection in large epidemiological studies.

## Introduction

Increased use of the Internet over the past decade provides new opportunities to use Web-based questionnaires in epidemiological studies. Compared to traditional paper-based questionnaires, Web-based alternatives may simplify data collection and improve the quality of data [1-3]. The need to design and validate questionnaires specifically for use on the Web has, therefore, increased in recent years. Many Web-based questionnaires used today are directly transferred from printed questionnaires rather than being originally developed for the medium in which they are used. Thus, few take advantage of the potential interactive features offered by the Web. For example, the burden on the respondent can be decreased by using follow-up questions, and errors can be minimized by implementing automatic controls for missing, inconsistent, or anomalous answers [4].

At present, there are few physical activity questionnaires available specifically developed for the Web. To the best of our knowledge, only one Web-based questionnaire (that assessed lifetime physical activity) has been validated and showed acceptable results [5]. Therefore, we developed a series of interactive questionnaires that assess total physical activity and inactivity in different age groups. The Active-Q questionnaire assesses physical activity and inactivity during the past year in subjects over 18 years.

The primary aim of this study was to test the validity of Active-Q against the doubly labeled water (DLW) method, the criterion standard for measuring energy expenditure [6]. Furthermore, the reproducibility of Active-Q was assessed by comparing the results obtained from the questionnaire on two separate occasions.

## Methods

### Study Participants

Study participants of both sexes, aged 20 to 65 years, were recruited in April of 2009 through public advertisements (including advertisements on the campuses of three universities) around Stockholm, Sweden. Participants were required to have an email address and access to the Internet. Exclusion criteria were any form of weight alteration diet, pregnancy, or having given birth during the ten months prior to the start of the study. Participants were provided with written and verbal information about the study. All participants gave their written informed consent prior to entering the study.

In total, 40 individuals were recruited. Data from three participants were excluded from the analysis because of illness during data collection, incomplete data from the first Active-Q questionnaire (ie, < 1 hour of leisure time activities per day reported), and unreliable DLW data, respectively. After exclusions, data from 37 participants remained for analysis.

### Study Design

**Figure 1:**
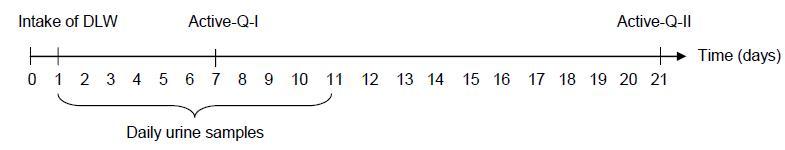
Timeline of study showing days that participants took doubly labeled water (DLW), provided urine samples, took the first questionnaire (Active-Q-I), and the second questionnaire (Active-Q-II).

Figure 1 illustrates the design of the study procedure. Participants filled out the Active-Q questionnaire on two occasions, separated by two weeks, and their energy expenditure was measured for 11 consecutive days using the DLW method [7]. On day 1, a baseline urine sample was collected before the participants drank a dose of DLW. Daily urine samples were collected for the next 10 consecutive days. Participants received the first Active-Q questionnaire (Active-Q-I ) 7 days after the start of the study; they received the second questionnaire (Active-Q-II) two weeks later. Questionnaires were distributed via email. Individual usernames and passwords served as unique identifiers in the Web-questionnaire program. Email reminders were sent to participants who had not responded to the questionnaire within two days of it being sent out. After responding to Active-Q-I, participants were asked to evaluate its user-friendliness and self-report their weight, height, level of education, and tobacco use.

The study was approved by the Research Ethics Committee at the Karolinska Institutet, Stockholm, Sweden.

### Active-Q

**Figure 2:**
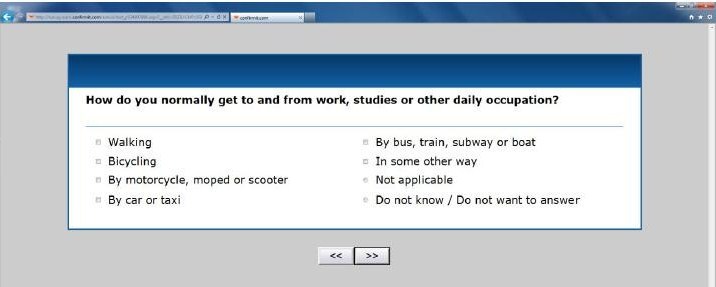
Screenshot of Active-Q screening question regarding mode of transportation to and from daily activities.

**Figure 3:**
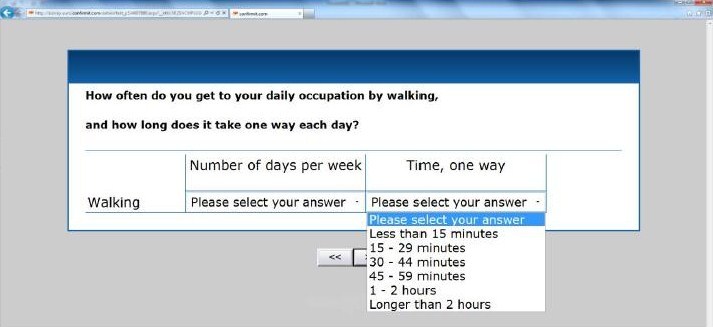
Screenshot of follow-up question for activities selected in screening question regarding transportation showing an example of possible answers.

Active-Q is a Web-based questionnaire designed to assess physical activity and inactivity in adults older than 18 years. Respondents are asked to report their habitual activity during the past year. Active-Q covers four different domains: (1) daily occupation, (2) transportation to and from daily occupation, (3) leisure time activities, and (4) regular sporting activities (see Multimedia Appendix 1). The leisure time domain includes activities such as housework, watching television, and using the computer. Within each domain, activities with similar intensity levels are grouped together as appropriate. Inactivity is assessed by questions about sedentary behavior during daily occupation, and by questions regarding time spent watching television and using a computer during leisure time. The initial questions within the means of transportation to and from daily occupation, leisure time activities, and sporting activities domains are screening questions that list all the activities included in the domain. The participant selects those activities within this list that he or she practices. Follow-up questions about frequency and duration pertain only to those activities selected in the screening question. With the exception of one question (the total number of hours spent in daily occupation), all questions have predefined answers regarding frequency and duration. Respondents report the frequency and duration of leisure time activities they perform at least once per week and sport activities they perform on a regular basis, thus limiting the number of questions for each respondent. Figure 2 shows screenshots of an initial screening question in Active-Q and Figure 3 shows a screenshot of a follow-up question for a selected activity regarding frequency and duration.

In total, Active-Q includes 35 questions with all activities linked to a corresponding Metabolic Equivalent Task (MET) value [8, 9]. Table 1 lists the activities included in Active-Q and their corresponding MET values.

Energy expenditure (EE) was estimated based on the assumption that 1 MET equals 1 kcal ∙ kg^-1^ ∙ h^-1^ [8]. To calculate the energy expenditure based on the results from Active-Q, MET values assigned to each activity were multiplied by the participants’ weight (kg), the average daily duration of the activity (h/d), and a conversion factor of 4.184, to transform the values from kcal to kJ:

EE_activity_ (kJ/day) = MET_activity_ ∙ Weight (kg) ∙ Duration_activity_ (h/d) ∙ 4.184

Total energy expenditure was expressed in terms of the crude total energy expenditure and the total energy expenditure per 24 hours. The crude total energy expenditure was obtained by summing the contributions of energy from each activity in Active-Q. The total energy expenditure over a 24-hour period was calculated by adding eight hours of sleep to the crude results from Active-Q. If the resulting time differed from 24 hours, time was added or subtracted to obtain the adjusted total energy expenditure per 24 hours. A MET value of 2.0 was assumed for the time added and subtracted [8, 10].

**Table 1:** Questions included in Active-Q^a^ and corresponding MET values.

Activity category		MET value
**Daily occupation^b^**		
	Mostly sitting	1.5
	A combination of sitting and standing up	2.3
	Mostly standing up	3.0
	Some physical activity	4.5
	Heavy manual labor	6.0
**Transportation**		
	Walking	4.0
	Bicycling	4.0
	By motorcycle or scooter	2.5
	By car or taxi	1.0
	By bus, train, subway, or boat	1.0
**Leisure time activities**		
	Watching TV/DVDs	1.0
	Using the computer	1.0
	Sitting listening to music, sewing, etc	1.0
	Playing a musical instrument or active computer games	2.0
	Doing household chores	3.0
	Shopping or other errands	2.3
	Dancing	3.0
	Walking	3.4
	Bicycling	8.0
**Regular sporting activities**		
	Aerobics	6.5
	Weight lifting	6.0
	Jogging or running	8.0
	Athletics	6.0
	Spinning	8.5
	Swimming	6.0
	Soccer, basketball, volleyball, or floorball (floor hockey)	6.0
	Golf	4.5
	Dance class	4.5
	Horseback riding	4.0
	Ice skating, ice hockey, or bandy	7.0
	Skiing (downhill or cross country)	7.0
	Martial arts	10.0
	Boxing or wrestling	6.0
	Tennis, badminton, or squash	7.0

	Table tennis	4.0
	Rowing, canoeing, surfing, or sailing	3.0
	Motor sports	4.0
	Rock climbing	8.0
	Other	2.5

^a^ All domain activities (ie, daily occupation, transportation, leisure time, and sporting activities), including frequency and duration, were assessed via an initial screening questionnaire.

^b^ Participants ranked their overall effort in this category on a scale from 1 to 5.

### Doubly Labeled Water

Doubly labeled water was used as the criterion measure of total energy expenditure [7] for 11 consecutive days. The logistics of the study prevented the preparation of individually tailored DLW doses. Instead, two standard doses were prepared adopting a strategy similar to that of Trabulsi et al [11]. A bulk dose of DLW was made by adding 44 g of^2^H_2_O (99.98% sterility tested, CK Gas Products Ltd, Hampshire, UK) to 1 L 10% normalized H_2_^18^O (SerCon Ltd, Cheshire, UK). Participants gave a baseline urine sample before drinking either 108 g (participants < 75 kg) or 141 g (participants ≥ 75 kg) of the bulk dose depending on their self-reported weight at the beginning of the study. In addition, participants collected 5 ml daily urine samples (excluding the first morning void) on each of the following 10 days. Participants recorded the date and time of each sample collection and kept the samples refrigerated until they were returned to the research team. The research team sent the samples to MRC Human Nutrition Research in Cambridge, UK, for analysis.

The principles for the slightly modified analyses of the isotopic enrichment in the samples have been described in detail in previous studies [12, 13]. Briefly, for^2^H analysis, 0.4 ml samples of undistilled urine were equilibrated with hydrogen gas using platinum catalyst rods to promote the rapid exchange between^2^H and^1^H. The 3.5-ml sample vials were first flush-filled with hydrogen, producing approximately 3 ml of gas at 1 bar atmosphere, and then equilibrated at 22.0 +0.1°C for 6 hours. Cryogenically dried hydrogen gas from the headspaces of each sample was analyzed using isotope ratio mass spectrometry (Sira10, VG Isogas, Winsford, Cheshire, UK).

Measurements of the^18^O/^16^O ratios were made using an AP2003 continuous-flow isotope ratio mass spectrometer (Analytical Precision Ltd, Northwich, Cheshire, UK). Urine samples of 0.5 ml were placed in 10 ml Vacutainers (Labco Ltd, High Wycombe, UK), flush-filled with 5% CO_2_ in nitrogen and then equilibrated on blood tube rotators overnight at room temperature before analysis. In all cases, analytical standards prepared in-house and traceable to the international standards, Vienna Standard Mean Ocean Water (VSMOW) and Standard Light Arctic Precipitation (SLAP), were included in each batch of samples analyzed.

Total energy expenditure was calculated as described by Schoeller et al [14] from the slopes and intercepts of the isotope disappearance curves using urine samples collected on days 1-3 and 8-10, respectively. The value of the respiratory quotient was assumed to be 0.85.

### Statistical Methods

Descriptive statistics were used to present the characteristics of the participants. Results are reported as mean values and standard deviations (SD), with the exception of time to respond to the questionnaire, which is presented as the median response time, and results of user-friendliness, which is presented in absolute numbers. For categorical variables or variables with a skewed distribution, Fisher’s exact test was performed to assess potential differences between men and women, and participants < 30 and ≥ 30 years of age. For those continuous variables typically normally distributed (eg, height, weight, and BMI), *t* tests were used to assess potential differences. The level of significance was set at *P* < .05.

The degree of association between the total energy expenditure obtained from Active-Q-I, both crude and adjusted to reflect a 24-hour day, and the DLW method, was assessed using Spearman correlation coefficients. Because Spearman correlation coefficients do not detect systematic differences between the methods, we used the Bland-Altman technique [15] to determine the absolute agreement. The difference in energy expenditure assessed from Active-Q-I (adjusted to reflect a 24-hour period) and DLW was plotted on the y-axis, and the mean values of the two assessments on the x-axis. The limits of agreement, equal to ±2 SD of the mean difference, provide a measure of the variation. The reproducibility of Active-Q was assessed using intraclass correlation coefficients [16]. The ANOVA estimator of intraclass correlation coefficients was computed. All analyses were performed using STATA 11.1 (STATA Corporation, College Station, TX).

## Results

Table 2 displays the baseline characteristics of the participants included in analysis. Subjects were predominantly female (> 80%) and under the age of 40 (> 70%). The self-reported height and weight resulted in a mean body mass index (BMI) of 23.0 (±3.8) kg/m^2^. Men were taller (*P* < .001) and weighed more (*P* < .001) than women. Subjects ≥ 30 years of age, weighed more (*P* = .024) and had a higher BMI (*P* = .007) compared with participants < 30 years of age. No other statistically significant differences were found between sex or age groups.

The median time required to fill out the Active-Q questionnaire on the first occasion was 6 minutes and 6 seconds. On average, the activities reported corresponded to 11 hours and 24 minutes of a typical day (excluding time spent sleeping). In the evaluation of the user-friendliness of the questionnaire, the majority of respondents (27/37, 73%) graded the questionnaire as “easy” or “very easy” to answer. Respondents were also asked to give the questionnaire an overall grade on a scale from 1 (worst) to 5 (best). The majority of respondents (21/37, 57%) graded the questionnaire a 4. An equal number of participants (7/37, 19%), graded the questionnaire a 3 or a 5. The remainder of participants (2/37, 5%) gave the questionnaire a 2. No respondents gave the questionnaire a 1, the worst possible grade. The mean overall grade for Active-Q-I was 3.9 ±0.8. Participants ≥ 30 years graded the questionnaire higher than younger participants (*P* = .006). No other statistically significant age-related or sex-related differences were seen in the results.

The mean total daily energy expenditure measured with DLW was 11,229 kJ (SD 2256), while the mean energy expenditure from Active-Q-I and Active-Q-II, adjusted to reflect a 24-hour period, was 11,667 kJ (SD 3212) and 11,529 kJ (SD 2758), respectively. The mean crude energy expenditure assessed with Active-Q-I was 7008 kJ (SD 3854). The crude energy expenditure for each domain of Active-Q-I was 2971 kJ (SD 1736) for daily occupation, 434 kJ (SD 388) for transportation, 2243 kJ (SD 1550) for leisure time activities, and 1360 kJ (SD 3044) for regular sporting activities. The mean crude energy expenditure from Active-Q-II was 6439 kJ (SD 2614). The crude energy expenditure for each domain of Active-Q-II was 3005 kJ (SD 1537) for daily occupation, 365 kJ (SD 296) for transportation, 2254 kJ (SD 1880) for leisure time activities, and 815 kJ (SD 761) for regular sporting activities.

The Spearman correlation coefficient between the crude total daily energy expenditure assessed with Active-Q-I and DLW was *r* = 0.42 (*P* = .01). After adjusting Active-Q-I for a 24-hour day, the Spearman correlation coefficient increased to *r* = 0.52 (*P* < .001).

**Figure 4:**
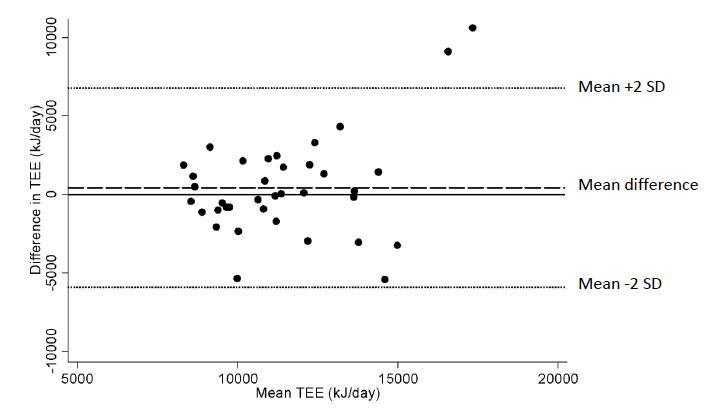
Bland-Altman plot illustrating the difference in total daily energy expenditure (TEE) between Active-Q-I (adjusted to reflect a 24-hour day) and the DLW method. The absolute difference in TEE between Active-Q-I and DLW is plotted on the y-axis and the mean of the two assessments on the x-axis. Each data point represents one participant (n= 37).

Figure 4 shows a Bland-Altman plot illustrating the differences in the total energy expenditure during 24 hours assessed with Active-Q-I and DLW. No clear trend (proportional error) can be seen in the plot. Most data points fall within the limits of agreement (±2 SD), although it should be noted that the limits of agreement were wide. The mean difference between the methods of assessment was 440 kJ, with Active-Q overestimating the total daily energy expenditure compared with DLW. According to the Bland-Altman plot, there were two outliers for which Active-Q noticeably overestimated total energy expenditure. One of the outliers reported extreme amounts of sporting activities in Active-Q, while the other had a BMI above 35 kg/m^2^.

The intraclass correlation coefficient for the crude total energy expenditure assessed from the questionnaire on two occasions was 0.66 (95% CI 0.47-0.84). After adjustments to a 24-hour day, the value increased to 0.83 (95% CI 0.73-0.93).

**Table 2:** Baseline characteristics of the study population (n = 37).

	n	(%)
**Sex**		
Female	30	(81)
Male	7	(19)
**Age (y)**		
< 30	22	(59)
30-39	5	(14)
40-49	5	(14)
50-59	4	(11)
> 60	1	(3)
**BMI (kg/m^2^)**		
< 20	3	(8)
20-25	28	(76)
> 25	6	(16)
**Education (y)**		
9-12	7	(19)
> 12	30	(81)
**Smoking status^a^**		
Current	2	(5)
Previous	8	(22)
Never	25	(68)
**Snuff use^a^**		
Current	4	(11)
Previous	6	(16)
Never	26	(70)
**Watching television^b^ (h/d)**		
< 1	25	(68)
1-3	10	(27)
≥ 3	2	(5)
**Computer use^b, c^ (h/d)**		
< 1	23	(62)
1-3	8	(22)
≥ 3	6	(16)
**Regular sport activities^b^ (h/d) **		
0	5	(14)
< 1	26	(70)
1-3	5	(14)
≥ 3	1	(3)

^a^ Percentages do not equal 100 because of missing data

^b^ Reported in Active-Q-I

^c^ During leisure time

## Discussion

The results of this study demonstrate that Active-Q is a user-friendly questionnaire that provides valid and reproducible estimates of total energy expenditure when compared with objective DLW measurements, the criterion standard [6].

The validity and reproducibility of many paper-based physical activity questionnaires in use today are low [17], although some show reasonable validity [18-22]. The number of questionnaires validated against DLW is limited and, according to two reviews, few studies have shown Spearman correlations above 0.50 [23, 24]. However, Besson et al [20] recently reported a correlation of 0.67. Nevertheless, all questionnaires previously validated against DLW have, to the best of our knowledge, been paper-based and not adapted for the Web, making comparisons with Active-Q difficult.

Important factors in making a questionnaire user-friendly are the length and the level of details in a questionnaire, plus the way in which questions are structured and the order in which they are presented [25]. It is also important to adapt the questionnaire to the medium in which it is to be used for data collection. Web-based questionnaires offer many advantages compared with traditional paper-based questionnaires. The Web allows the use of skip and follow-up patterns, thereby increasing the user-friendliness by creating interactivity, which may reduce dropout [3, 26]. Technical features, such as checking the plausibility of answers, decrease the measurement error [27]. Computerized administration also means reduced costs for data collection and analysis, and facilitates the administration of large studies [1, 26]. Active-Q is interactive and takes advantage of follow-up questions with predefined answers, which minimizes the time required to answer the questions and decreases the risk of mistyping. Further, checks for missing answers are implemented in Active-Q, and respondents are prevented from proceeding to the next question in the questionnaire before answering the previous questions.

Today, more than 90% of the population in Sweden between 16 and 74 years of age have access to the Internet at home [28]. Therefore, we do not believe that concerns raised previously regarding high non-response rates and the introduction of selection bias will be a problem when collecting Web-based data in Sweden [26]. However, it is difficult to draw any conclusions regarding non-response rates and selection bias from this study, as a pre-requisite for participation was access to the Internet and an email address. A previous study comparing response rates to Web- and paper-based dietary questionnaires in Sweden reported that the response rate for paper questionnaires was approximately 15% higher than for Web questionnaires [3]. However, this study was performed in 2002 and access to, and the use of, the Internet have increased considerably in Sweden during the past decade [28].

Among Swedish individuals between 16 and 44 years of age, over 97% reported to be frequent users of the Internet compared to 90% for individuals between 45 and 54 years, and 70% for individuals between 55 and 74 years [28]. Although younger individuals are more frequent users of the Internet, the vast majority of the older individuals also use the Internet regularly. Therefore, we believe that a Web-based questionnaire is suitable for a wide age range. However, a larger validation study would clarify how applicable a Web-based questionnaire would be across different age groups.

Ideally, in validation studies, the reference method should reflect the same period of time as the new method being validated. However, habitual physical activity was assessed over a one-year period with Active-Q, while that measured with the DLW method reflects physical activity over 11 days. Repeated reference measures of DLW over a longer period of time would have been preferable, but this was neither practical nor possible in our study setting. Another drawback of this study is that we were only able to validate measures of total energy expenditure. Because the DLW method does not discriminate between different levels of intensity in activity, we were not able to validate Active-Q in this respect. Since different aspects of activity may affect health-related outcomes differently, the ability of Active-Q to accurately assess this needs to be validated in future studies using, for example, accelerometers.

When estimating the total daily energy expenditure with Active-Q, we assumed that all participants slept for eight hours [29]. The true variation in the duration of sleep among individuals may reduce the accuracy of results, leading to an underestimation of validity of Active-Q. In future studies, a question about the average duration of sleep will be added to Active-Q. On average, the participants’ reported total activity time in Active-Q-I was in line with other studies on the Swedish population [30]. However, even after adjusting for sleep, most participants underestimated the time spent in daily activities in Active-Q, whereas a small proportion overestimated it. Therefore, results were adjusted by adding or subtracting under- or over-reported time to obtain a total of 24 hours in order to make the data comparable to the average total daily energy expenditure obtained from DLW measurements. Missing and overestimated time was adjusted by using a MET value of 2.0. Missing time was assumed to reflect a combination of common activities, such as self-care and preparing and eating meals, and overestimated time to reflect an average MET value of activities included in Active-Q.

The use of published MET values [9] to represent the energy cost for a specific activity constitutes a limitation in most physical activity questionnaires. The MET value for a specific activity assumes the same energy expenditure per kilogram of body weight for all individuals, regardless of variations in both mechanical and metabolic efficiency [8, 9], thereby leading to the risk of potential misclassification in assessments of energy expenditure. However, the measure of physical activity used in many epidemiological studies is that of MET hours, avoiding the issue of weight being a factor affecting the final outcome in terms of energy expenditure, which is obtained by multiplying MET hours with participants’ weight. Hence, the exact energy expenditure of participants is less important than ranking individuals correctly.

While the point estimate of the intraclass correlation for the adjusted total energy expenditure assessed with Active-Q is high, the point estimate for the assessed crude total energy expenditure is lower, and the confidence intervals around both estimates are rather wide. The wide confidence intervals, and the rather low point estimate obtained for the crude total energy expenditure, might be explained by our small sample size and sampling variability. To obtain more certain measures of reproducibility, we need to conduct a larger study to assess reproducibility of both assessments of total energy expenditure, as well as energy expenditure within each individual domain of Active-Q.

Our study population was recruited from the Stockholm area, including the campuses of three universities, and the participants were young and predominantly female. This may limit the generalizability of our results. The overall level of education among the participants was high, with more than 80% having an education longer than 12 years, compared with 36% in the general Swedish population aged 16-64 years [31]. Higher education has been associated with more accurate recall of physical activity [32]; therefore, our results may be biased in this direction. In addition, bias due to self-selection of participants may be present because recruitment was voluntary through public advertisements. Health-conscious individuals may be more prone to participate in a study on physical activity.

To the best of our knowledge, Active-Q is one of the first validated physical activity questionnaires specifically designed for Web-based use. Despite the limitations mentioned previously, we believe Active-Q to be a good assessment method in studies collecting Web-based data. Active-Q is currently in use in three large ongoing cohort studies in Sweden, aimed at recruiting hundreds of thousands of people [33].

An increasing number of studies are relying on the collection of data via the Web, and there is a need for valid Web-based methods of assessing physical activity. We have demonstrated that Active-Q is a valid method for estimating total energy expenditure. Furthermore, Active-Q is also a reproducible and user-friendly method. We conclude that Active-Q is a suitable method for collecting Web-based data on physical activity and inactivity in large epidemiological studies.

## Multimedia Appendix 1

The Active-Q Physical Activity Questionnniare.

## Abbreviations

BMI: body mass index
DLW: doubly labeled water
EE: energy expenditure
MET: metabolic equivalent task
SLAP: standard light arctic precipitation
VSMOW: Vienna standard mean ocean water

## References

[1] Ekman A, Litton JE (2007). New times, new needs; e-epidemiology. Eur J Epidemiol.

[2] Ekman A, Dickman PW, Klint A, Weiderpass E, Litton JE (2006). Feasibility of using web-based questionnaires in large population-based epidemiological studies. Eur J Epidemiol.

[3] Bälter KA, Bälter O, Fondell E, Lagerros YT (2005). Web-based and mailed questionnaires: a comparison of response rates and compliance. Epidemiology.

[4] Touvier M, Méjean C, Kesse-Guyot E, Pollet C, Malon A, Castetbon K, Hercberg S (2010). Comparison between web-based and paper versions of a self-administered anthropometric questionnaire. Eur J Epidemiol.

[5] De Vera MA, Ratzlaff C, Doerfling P, Kopec J (2010). Reliability and validity of an internet-based questionnaire measuring lifetime physical activity. Am J Epidemiol.

[6] Ainslie P, Reilly T, Westerterp K (2003). Estimating human energy expenditure: a review of techniques with particular reference to doubly labelled water. Sports Med.

[7] Davidsson L (2009). Assessment of body composition and total energy expenditure in humans using stable isotope techniques. IAEA Human Health Series.

[8] Ainsworth BE, Haskell WL, Leon AS, Jacobs DR, Montoye HJ, Sallis JF, Paffenbarger RS (1993). Compendium of physical activities: classification of energy costs of human physical activities. Med Sci Sports Exerc.

[9] Ainsworth BE, Haskell WL, Whitt MC, Irwin ML, Swartz AM, Strath SJ, O'Brien WL, Bassett DR, Schmitz KH, Emplaincourt PO, Jacobs DR, Leon AS (2000). Compendium of physical activities: an update of activity codes and MET intensities. Med Sci Sports Exerc.

[10] Norman A, Bellocco R, Bergström A, Wolk A (2001). Validity and reproducibility of self-reported total physical activity--differences by relative weight. Int J Obes Relat Metab Disord.

[11] Trabulsi J, Troiano RP, Subar AF, Sharbaugh C, Kipnis V, Schatzkin A, Schoeller DA (2003). Precision of the doubly labeled water method in a large-scale application: evaluation of a streamlined-dosing protocol in the Observing Protein and Energy Nutrition (OPEN) study. Eur J Clin Nutr.

[12] Assah FK, Ekelund U, Brage S, Wright A, Mbanya JC, Wareham NJ (2011). Accuracy and validity of a combined heart rate and motion sensor for the measurement of free-living physical activity energy expenditure in adults in Cameroon. Int J Epidemiol.

[13] Bluck LJC (2008). Doubly labelled water for the measurement of total energy expenditure in man - progress and applications in the last decade. Nutrition Bulletin.

[14] Schoeller DA, Ravussin E, Schutz Y, Acheson KJ, Baertschi P, Jéquier E (1986). Energy expenditure by doubly labeled water: validation in humans and proposed calculation. Am J Physiol.

[15] Bland JM, Altman DG (1986). Statistical methods for assessing agreement between two methods of clinical measurement. Lancet.

[16] Lee J, Koh D, Ong CN (1989). Statistical evaluation of agreement between two methods for measuring a quantitative variable. Comput Biol Med.

[17] Westerterp KR (2009). Assessment of physical activity: a critical appraisal. Eur J Appl Physiol.

[18] Conway JM, Irwin ML, Ainsworth BE (2002). Estimating energy expenditure from the Minnesota Leisure Time Physical Activity and Tecumseh Occupational Activity questionnaires - a doubly labeled water validation. J Clin Epidemiol.

[19] Craig CL, Marshall AL, Sjöström M, Bauman AE, Booth ML, Ainsworth BE, Pratt M, Ekelund U, Yngve A, Sallis JF, Oja P (2003). International physical activity questionnaire: 12-country reliability and validity. Med Sci Sports Exerc.

[20] Besson H, Brage S, Jakes RW, Ekelund U, Wareham NJ (2010). Estimating physical activity energy expenditure, sedentary time, and physical activity intensity by self-report in adults. Am J Clin Nutr.

[21] Staten LK, Taren DL, Howell WH, Tobar M, Poehlman ET, Hill A, Reid PM, Ritenbaugh C (2001). Validation of the Arizona Activity Frequency Questionnaire using doubly labeled water. Med Sci Sports Exerc.

[22] Pettee Gabriel K, McClain JJ, Schmid KK, Storti KL, Ainsworth BE (2010). Reliability and convergent validity of the past-week Modifiable Activity Questionnaire. Public Health Nutr.

[23] Neilson HK, Robson PJ, Friedenreich CM, Csizmadi I (2008). Estimating activity energy expenditure: how valid are physical activity questionnaires?. Am J Clin Nutr.

[24] van Poppel MN, Chinapaw MJ, Mokkink LB, van Mechelen W, Terwee CB (2010). Physical activity questionnaires for adults: a systematic review of measurement properties. Sports Med.

[25] Jacobs DR, Ainsworth BE, Hartman TJ, Leon AS (1993). A simultaneous evaluation of 10 commonly used physical activity questionnaires. Med Sci Sports Exerc.

[26] Russell CW, Boggs DA, Palmer JR, Rosenberg L (2010). Use of a web-based questionnaire in the Black Women's Health Study. Am J Epidemiol.

[27] van Gelder MM, Bretveld RW, Roeleveld N (2010). Web-based questionnaires: the future in epidemiology?. Am J Epidemiol.

[28] Arrhenius C, Ewerdahl D (2011). Statistics Sweden.

[29] Rydenstam K (2003). Statistics Sweden.

[30] Lagerros YT, Bellocco R, Adami HO, Nyrén O (2009). Measures of physical activity and their correlates: the Swedish National March Cohort. Eur J Epidemiol.

[31] Sundh L (2010). Statistics Sweden.

[32] Falkner KL, McCann SE, Trevisan M (2001). Participant characteristics and quality of recall of physical activity in the distant past. Am J Epidemiol.

[33] Almqvist C, Adami HO, Franks PW, Groop L, Ingelsson E, Kere J, Lissner L, Litton JE, Maeurer M, Michaëlsson K, Palmgren J, Pershagen G, Ploner A, Sullivan PF, Tybring G, Pedersen NL (2011). LifeGene--a large prospective population-based study of global relevance. Eur J Epidemiol.

