# Supplementary material for: Active-Q: Validation of the Web-Based Physical Activity Questionnaire Using Doubly Labeled Water
Source: J Med Internet Res. 2012 Feb 15;14(1):e29. doi: 10.2196/jmir.1974 (PMC3374527; doi:10.2196/jmir.1974)
Supplement: Supplementary file 1 [file jmir_v14i1e29_app1.pdf]

Active-Q – A Physical Activity Questionnaire for Adults

The following questions concern your physical activity during the last year

Physical activity level – daily occupation

What is your normal activity level at your daily occupation (work, studies or equivalent)?

Mostly Sitting	A combination of sitting and standing up	Mostly standing up	Some physical activity	Heavy manual labor
☐	☐	☐	☐	☐

Duration – daily occupation

Approximately how many hours per week do you normally conduct your daily occupation (work, studies or equivalent)?

❍ 5 hours or less
❍ 6 - 10 hours
❍ 11 – 20 hours
❍ 21 – 30 hours
❍ 31 – 40 hours
❍ 41 – 50 hours
❍ More than 50 hours
❍ Don't know / Don't want to answer

Means of transportation to daily occupation

How do you normally get to and from work, studies or other daily occupation?

❑ Walking
❑ Cycling
❑ By motorcycle, moped or scooter
❑ By car or taxi
❑ By bus, train, subway or boat
❑ Other
❍ Does not apply to me
❍ Don't know / Don't want to answer

Follow-up questions regarding means of transportation.

Selected activities in the previous screening question are presented to the respondent

How often do you get to your daily occupation in the following ways and approximately how long does it take?

	Number of days per week	Time, one way per day
Walking	❍ 1<br>❍ 2<br>❍ 3<br>❍ 4<br>❍ 5<br>❍ 6<br>❍ 7	❍ Less than 15 minutes<br>❍ 15 - 29 minutes<br>❍ 30 - 44 minutes<br>❍ 45 - 59 minutes<br>❍ 1 - 2 hours<br>❍ Longer than 2 hours

	Number of days per week	Time, one way per day
Cycling	❍ 1<br>❍ 2<br>❍ 3<br>❍ 4<br>❍ 5<br>❍ 6<br>❍ 7	❍ Less than 15 minutes<br>❍ 15 - 29 minutes<br>❍ 30 - 44 minutes<br>❍ 45 - 59 minutes<br>❍ 1 - 2 hours<br>❍ Longer than 2 hours
By motorcycle, moped or scooter	❍ 1<br>❍ 2<br>❍ 3<br>❍ 4<br>❍ 5<br>❍ 6<br>❍ 7	❍ Less than 15 minutes<br>❍ 15 - 29 minutes<br>❍ 30 - 44 minutes<br>❍ 45 - 59 minutes<br>❍ 1 - 2 hours<br>❍ Longer than 2 hours
By car or taxi	❍ 1<br>❍ 2<br>❍ 3<br>❍ 4<br>❍ 5<br>❍ 6<br>❍ 7	❍ Less than 15 minutes<br>❍ 15 - 29 minutes<br>❍ 30 - 44 minutes<br>❍ 45 - 59 minutes<br>❍ 1 - 2 hours<br>❍ Longer than 2 hours
By bus, train, subway or boat	❍ 1<br>❍ 2<br>❍ 3<br>❍ 4<br>❍ 5<br>❍ 6<br>❍ 7	❍ Less than 15 minutes<br>❍ 15 - 29 minutes<br>❍ 30 - 44 minutes<br>❍ 45 - 59 minutes<br>❍ 1 - 2 hours<br>❍ Longer than 2 hours
Other	❍ 1<br>❍ 2<br>❍ 3<br>❍ 4<br>❍ 5<br>❍ 6<br>❍ 7	❍ Less than 15 minutes<br>❍ 15 - 29 minutes<br>❍ 30 - 44 minutes<br>❍ 45 - 59 minutes<br>❍ 1 - 2 hours<br>❍ Longer than 2 hours

Leisure time activities

Which of the following activities do you engage in at least once per week in your leisure time?

❑ Watching TV/DVDs etc.
❑ Using the computer, reading emails, playing computer games, play station, X-box etc.
❑ Sitting reading, writing, sewing etc.
❑ Playing a musical instrument or active computer games (e.g. Wii)
❑ Doing household chores (e.g. cleaning, laundry, child care, gardening etc.)
❑ Shopping, or other errands
❑ Dancing
❑ Walking, Nordic walking, walking a dog (not as transport to daily occupation)
❑ Bicycling
(not as transport to daily occupation)
❍ None of the above
❍ Don’t know /Don’t want to answer

Follow-up questions regarding leisure time activities.

Selected activities in the previous screening question are presented to the respondent

How many days per week do you dedicate time to the following leisure time activities and how much time do you spend per day on the activity?

	Number of days per week	Total time per day
Watching TV/DVDs	❍ 1<br>❍ 2<br>❍ 3<br>❍ 4<br>❍ 5<br>❍ 6<br>❍ 7	❍ Less than 30 minutes<br>❍ 30 - 59 minutes<br>❍ 1 - 2 hours<br>❍ 3 - 4 hours<br>❍ 5 - 8 hours<br>❍ Longer than 8 hours
Using the computer	❍ 1<br>❍ 2<br>❍ 3<br>❍ 4<br>❍ 5<br>❍ 6<br>❍ 7	❍ Less than 30 minutes<br>❍ 30 - 59 minutes<br>❍ 1 - 2 hours<br>❍ 3 - 4 hours<br>❍ 5 - 8 hours<br>❍ Longer than 8 hours
Sitting writing, reading, sewing etc.	❍ 1<br>❍ 2<br>❍ 3<br>❍ 4<br>❍ 5<br>❍ 6<br>❍ 7	❍ Less than 30 minutes<br>❍ 30 - 59 minutes<br>❍ 1 - 2 hours<br>❍ 3 - 4 hours<br>❍ 5 - 8 hours<br>❍ Longer than 8 hours]
Playing a musical instrument or active computer games (e.g. Wii)	❍ 1<br>❍ 2<br>❍ 3<br>❍ 4<br>❍ 5<br>❍ 6<br>❍ 7	❍ Less than 30 minutes<br>❍ 30 - 59 minutes<br>❍ 1 - 2 hours<br>❍ 3 - 4 hours<br>❍ 5 - 8 hours<br>Longer than 8 hours
Doing household chores, cleaning, doing laundry, taking care of children, garden work etc.	❍ 1<br>❍ 2<br>❍ 3<br>❍ 4<br>❍ 5<br>❍ 6<br>❍ 7	❍ Less than 30 minutes<br>❍ 30 - 59 minutes<br>❍ 1 - 2 hours<br>❍ 3 - 4 hours<br>❍ 5 - 8 hours<br>Longer than 8 hours
Shopping or other errands	❍ 1<br>❍ 2<br>❍ 3<br>❍ 4<br>❍ 5<br>❍ 6<br>❍ 7	❍ Less than 30 minutes<br>❍ 30 - 59 minutes<br>❍ 1 - 2 hours<br>❍ 3 - 4 hours<br>❍ 5 - 8 hours<br>❍ Longer than 8 hours

	Number of days per week	Total time per day
Dancing (e.g. disco or ball room)	❍ 1<br>❍ 2<br>❍ 3<br>❍ 4<br>❍ 5<br>❍ 6<br>❍ 7	❍ Less than 30 minutes<br>❍ 30 - 59 minutes<br>❍ 1 - 2 hours<br>❍ 3 - 4 hours<br>❍ 5 - 8 hours<br>❍ Longer than 8 hours
Walking (not as transport to daily occupation), Nordic walking or walking a dog	❍ 1<br>❍ 2<br>❍ 3<br>❍ 4<br>❍ 5<br>❍ 6<br>❍ 7	❍ Less than 15 minutes<br>❍ 15 – 29 minutes<br>❍ 30 – 44 minutes<br>❍ 45 – 59 minutes<br>❍ 1 – 2 hours<br>❍ Longer than 2 hours
Cycling (not as transport to daily occupation)	❍ 1<br>❍ 2<br>❍ 3<br>❍ 4<br>❍ 5<br>❍ 6<br>❍ 7	❍ Less than 15 minutes<br>❍ 15 – 29 minutes<br>❍ 30 – 44 minutes<br>❍ 45 – 59 minutes<br>❍ 1 – 2 hours<br>❍ Longer than 2 hours

Overall sporting activity or exercise

Do you exercise or participate in any sporting activities regularly?

❍ Yes
❍ No
❍ Don’t know / Don’t want to answer

Sporting activities

What kind of exercise or sports activities do you participate in? If your type of activity is not included in the list below, please choose the most similar one.

❑ Aerobics or cardio fitness class
❑ Weight lifting
❑ Jogging, running or orienteering
❑ Athletics (e.g. high jump, long jump or three-step)
❑ Spinning or cycling in demanding terrain
❑ Swimming
❑ Ball sports (e.g. soccer, basketball, volley ball or floorball)
❑ Golf

❑ Dance class or competitive dancing (e.g. ballroom, ballet, jazz or street)
❑ Horseback riding
❑ Skating, ice hockey or bandy
❑ Skiing downhill or cross country
❑ Martial arts (e.g. judo or karate)
❑ Boxing or wrestling
❑ Tennis, badminton or table tennis
❑ Squash
❑ Sailing, surfing, canoeing or rowing
❑ Motor sports (e.g. motocross)
❑ Rock climbing
❑ Yoga, Pilates or Tai chi
❑ Other activity or sport (please state)____________
❍ None of these
❍ Don't know / Don't want to answer

Follow-up questions regarding regular sporting activities
Selected activities in the previous screening question are presented to the respondent

How often do you participate in the following sports or the following activities and for how long each time?

	Number of times	Time per session
Aerobics or cardio fitness class	❍ 1 - 3 times a month<br>❍ Once a week<br>❍ 2 - 3 times a week<br>❍ 4 - 5 times a week<br>❍ 6 - 7 times a week	❍ Less than 30 minutes<br>❍ 30 - 59 minutes<br>❍ 60 - 119 minutes<br>❍ 2 - 4 hours<br>❍ More than 4 hours
Weight lifting	❍ 1 - 3 times a month<br>❍ Once a week<br>❍ 2 - 3 times a week<br>❍ 4 - 5 times a week<br>❍ 6 - 7 times a week	❍ Less than 30 minutes<br>❍ 30 - 59 minutes<br>❍ 60 - 119 minutes<br>❍ 2 - 4 hours<br>❍ More than 4 hours
Jogging, running or orienteering	❍ 1 - 3 times a month<br>❍ Once a week<br>❍ 2 - 3 times a week<br>❍ 4 - 5 times a week<br>❍ 6 - 7 times a week	❍ Less than 30 minutes<br>❍ 30 - 59 minutes<br>❍ 60 - 119 minutes<br>❍ 2 - 4 hours<br>❍ More than 4 hours
Athletics (e.g. high jump, long jump or three-step)	❍ 1 - 3 times a month<br>❍ Once a week<br>❍ 2 - 3 times a week<br>❍ 4 - 5 times a week<br>6 - 7 times a week	❍ Less than 30 minutes<br>❍ 30 - 59 minutes<br>❍ 60 - 119 minutes<br>❍ 2 - 4 hours<br>More than 4 hours
Spinning or cycling in demanding terrain	❍ 1 - 3 times a month<br>❍ Once a week<br>❍ 2 - 3 times a week<br>❍ 4 - 5 times a week<br>6 - 7 times a week	❍ Less than 30 minutes<br>❍ 30 - 59 minutes<br>❍ 60 - 119 minutes<br>❍ 2 - 4 hours<br>More than 4 hours
Swimming	❍ 1 - 3 times a month<br>❍ Once a week	❍ Less than 30 minutes<br>❍ 30 - 59 minutes

	Number of times	Time per session
	❍ 2 - 3 times a week<br>❍ 4 - 5 times a week<br>6 - 7 times a week	❍ 60 - 119 minutes<br>❍ 2 - 4 hours<br>More than 4 hours
Ball sports (e.g. soccer, basketball, volley ball or floor ball)	❍ 1 - 3 times a month<br>❍ Once a week<br>❍ 2 - 3 times a week<br>❍ 4 - 5 times a week<br>❍ 6 - 7 times a week	❍ Less than 30 minutes<br>❍ 30 - 59 minutes<br>❍ 60 - 119 minutes<br>❍ 2 - 4 hours<br>More than 4 hours
Golf	❍ 1 - 3 times a month<br>❍ Once a week<br>❍ 2 - 3 times a week<br>❍ 4 - 5 times a week<br>❍ 6 - 7 times a week	❍ Less than 30 minutes<br>❍ 30 - 59 minutes<br>❍ 60 - 119 minutes<br>❍ 2 - 4 hours<br>❍ More than 4 hours
Dance class or competitive dancing	❍ 1 - 3 times a month<br>❍ Once a week<br>❍ 2 - 3 times a week<br>❍ 4 - 5 times a week<br>❍ 6 - 7 times a week	❍ Less than 30 minutes<br>❍ 30 - 59 minutes<br>❍ 60 - 119 minutes<br>❍ 2 - 4 hours<br>❍ More than 4 hours
Horseback riding	❍ 1 - 3 times a month<br>❍ Once a week<br>❍ 2 - 3 times a week<br>❍ 4 - 5 times a week<br>❍ 6 - 7 times a week	❍ Less than 30 minutes<br>❍ 30 - 59 minutes<br>❍ 60 - 119 minutes<br>❍ 2 - 4 hours<br>More than 4 hours
Skating, ice-hockey or bandy	❍ 1 - 3 times a month<br>❍ Once a week<br>❍ 2 - 3 times a week<br>❍ 4 - 5 times a week<br>❍ 6 - 7 times a week	❍ Less than 30 minutes<br>❍ 30 - 59 minutes<br>❍ 60 - 119 minutes<br>❍ 2 - 4 hours<br>More than 4 hours
Skiing, downhill or cross country	❍ 1 - 3 times a month<br>❍ Once a week<br>❍ 2 - 3 times a week<br>❍ 4 - 5 times a week<br>❍ 6 - 7 times a week	❍ Less than 30 minutes<br>❍ 30 - 59 minutes<br>❍ 60 - 119 minutes<br>❍ 2 - 4 hours<br>❍ More than 4 hours
Martial arts (e.g. judo or karate)	❍ 1 - 3 times a month<br>❍ Once a week<br>❍ 2 - 3 times a week<br>❍ 4 - 5 times a week<br>❍ 6 - 7 times a week	❍ Less than 30 minutes<br>❍ 30 - 59 minutes<br>❍ 60 - 119 minutes<br>❍ 2 - 4 hours<br>❍ More than 4 hours
Boxing or wrestling	❍ 1 - 3 times a month<br>❍ Once a week<br>❍ 2 - 3 times a week<br>❍ 4 - 5 times a week<br>❍ 6 - 7 times a week	❍ Less than 30 minutes<br>❍ 30 - 59 minutes<br>❍ 60 - 119 minutes<br>❍ 2 - 4 hours<br>❍ More than 4 hours
Tennis, badminton or table tennis	❍ 1 - 3 times a month<br>❍ Once a week<br>❍ 2 - 3 times a week<br>❍ 4 - 5 times a week<br>❍ 6 - 7 times a week	❍ Less than 30 minutes<br>❍ 30 - 59 minutes<br>❍ 60 - 119 minutes<br>❍ 2 - 4 hours<br>❍ More than 4 hours
Squash	❍ 1 - 3 times a month	❍ Less than 30 minutes

	Number of times	Time per session
	❍ Once a week<br>❍ 2 - 3 times a week<br>❍ 4 - 5 times a week<br>❍ 6 - 7 times a week	❍ 30 - 59 minutes<br>❍ 60 - 119 minutes<br>❍ 2 - 4 hours<br>❍ More than 4 hours
Sailing, surfing, canoeing or rowing	❍ 1 - 3 times a month<br>❍ Once a week<br>❍ 2 - 3 times a week<br>❍ 4 - 5 times a week<br>❍ 6 - 7 times a week	❍ Less than 30 minutes<br>❍ 30 - 59 minutes<br>❍ 60 - 119 minutes<br>❍ 2 - 4 hours<br>❍ More than 4 hours
Motor sports (e.g. motocross)	❍ 1 - 3 times a month<br>❍ Once a week<br>❍ 2 - 3 times a week<br>❍ 4 - 5 times a week<br>❍ 6 - 7 times a week	❍ Less than 30 minutes<br>❍ 30 - 59 minutes<br>❍ 60 - 119 minutes<br>❍ 2 - 4 hours<br>❍ More than 4 hours
Rock climbing	❍ 1 - 3 times a month<br>❍ Once a week<br>❍ 2 - 3 times a week<br>❍ 4 - 5 times a week<br>❍ 6 - 7 times a week	❍ Less than 30 minutes<br>❍ 30 - 59 minutes<br>❍ 60 - 119 minutes<br>❍ 2 - 4 hours<br>❍ More than 4 hours
Yoga, Pilates or Tai chi	❍ 1 - 3 times a month<br>❍ Once a week<br>❍ 2 - 3 times a week<br>❍ 4 - 5 times a week<br>❍ 6 - 7 times a week	❍ Less than 30 minutes<br>❍ 30 - 59 minutes<br>❍ 60 - 119 minutes<br>❍ 2 - 4 hours<br>❍ More than 4 hours
Other (displayed automatically)	❍ 1 - 3 times a month<br>❍ Once a week<br>❍ 2 - 3 times a week<br>❍ 4 - 5 times a week<br>❍ 6 - 7 times a week	❍ Less than 30 minutes<br>❍ 30 - 59 minutes<br>❍ 60 - 119 minutes<br>❍ 2 - 4 hours<br>❍ More than 4 hours

Do you compete regularly in a sport?
Follow-up question if respondent reported regular physical activity

❍ Yes
❍ No
❍ Don’t know / Don’t want to answer

How many hours do you usually sleep during a typical night?

❍ Less than 5 hours
❍ 6 hours
❍ 7 hours
❍ 8 hours
❍ 9 hours
❍ 10 hours or longer
❍ Don’t know / Don’t want to answer
